# The Crisis of Medical Education in the Arab World: A Student’s Perspective

**DOI:** 10.7759/cureus.39943

**Published:** 2023-06-04

**Authors:** Mohammad Abu-Jeyyab, Sallam Alrosan

**Affiliations:** 1 School of Medicine, Mutah University, Al-Karak, JOR; 2 Internal Medicine Department, Saint Luke’s Health System, Kansas City, USA

**Keywords:** education future, student's perspective, arab world, medical education curriculum, global healthcare systems

## Abstract

Medical education is an important and ever-changing profession that determines the future of healthcare and public health in any nation. It is also a complicated and difficult process that needs ongoing adaptation and innovation in order to satisfy the changing demands and expectations of health systems and communities. However, several challenges and limits impede the growth and quality of medical education in the Arab world, preventing it from reaching its full potential. In this article, we will highlight some of the major difficulties affecting medical education in the Arab world from our own experience as a medical student in one of the Arab nations.

## Editorial

The puzzle and the answer

The Educational Subjects

In the Arab world, there is a misalignment between the curriculum and the demands of local health systems and communities. Many medical schools in the area confront this difficulty, since they frequently follow outmoded or imported curricula that do not represent the epidemiological, cultural, and socioeconomic realities of the region [1]. This creates an imbalance between what is taught and what is required in practice, reducing the relevance and efficacy of medical education [1].

This gap is caused by a number of factors, including the rapid changes in the health landscape caused by the increasing burden of chronic diseases, aging, and environmental issues, the limited involvement of stakeholders in the design and evaluation of curricula, such as health professionals, policymakers, patients, and communities, the lack of integration of scientific research skills and evidence-based practice into curricula, and the insufficient use of innovative teaching methods [2].

Despite the challenges, the Arab world has seen some achievements and opportunities for improvement, such as the establishment of prestigious and reputable universities that provide medical education in accordance with the most recent and advanced educational curricula, the attraction of international partnerships and collaborations that enhance the exchange of expertise and resources, and the adoption of continuing medical education as a principle [2,3].

Faculty Members

One of the primary issues that medical education faces in the Arab world is a lack of skilled and competent faculty members and mentors who can provide high-quality education and training to medical students. This scarcity has an adverse effect on the quality and outcomes of medical education, as well as on the healthcare system and society as a whole. There are several reasons for this shortage, including the low attractiveness and retention of medical faculty members, a lack of incentives and recognition for teaching excellence and innovation among faculty members, a lack of training and development opportunities for faculty members to improve their teaching skills and knowledge in line with current advances and standards in their fields, and the high workload and stress that faculty members face in their academic and clinical roles, which often leads to burnout and dissatisfaction [4].

There are various viable remedies that might be applied by medical schools, hospitals, and policymakers throughout the Arab world to solve this dilemma. Some of these solutions include: increasing faculty recruitment and retention in the medical field by offering competitive salaries, benefits, and career opportunities that match their qualifications and competencies, rewarding and recognizing faculty members who demonstrate teaching excellence and innovation in their fields through awards, grants, promotions, and other forms of recognition, providing regular and effective training and development [5], and lowering faculty members' workloads and stress by maintaining a fair allocation of academic and clinical tasks, as well as providing them with enough support and tools to deal with their issues [6].

Limited Opportunities for Scientific Research and Innovation

Scientific research and innovation are critical in every civilization for expanding knowledge and improving health outcomes. However, the Arab world's medical education confronts several problems that hinder its ability to conduct high-quality research and create an innovative culture. Lack of finance, inadequate infrastructure, insufficient human resources, low academic standards, poor cooperation, and restricted dissemination of research findings are some of the obstacles [7].

Despite these obstacles, there are some possibilities and initiatives throughout the Arab world to improve research and innovation in medical education. Some institutions, for example, have developed centers for entrepreneurial studies, social innovation, and technology transfer to encourage faculty and student innovation and entrepreneurship [7]. To support research cooperation and innovation, several nations have also invested in science and technology parks and digital health platforms [8]. Furthermore, certain regional and international organizations have offered money and training programs to enhance the Arab world's research capacity building and knowledge sharing [7].

Infrastructure and Resources

Medical education in the Arab world is suffering from a significant lack of facilities and teaching and learning tools. The physical buildings, equipment, technology, and communication systems that enable the delivery of medical education are referred to as infrastructure. The human, financial, and material inputs that enable the execution of medical education programs are referred to as resources. According to a recent assessment, many Arab medical schools lack enough facilities and resources to achieve quality and accrediting criteria [7].

In the Arab world, limited facilities and resources have a detrimental influence on the quality and results of medical education. Inadequate infrastructure, for example, may limit the availability and accessibility of clinical training facilities, simulation labs, libraries, and research institutes [8]. Inadequate resources may have an impact on the recruitment and retention of talented academics, staff, and students, as well as the availability of scholarships, grants, and incentives. Furthermore, insufficient infrastructure and resources may stymie the implementation of novel teaching and learning approaches like problem-based learning, blended learning, and interprofessional education [9].

Several solutions have been suggested and executed by various parties to solve the Arab world's poor infrastructure and resources for medical education. Increased governmental and corporate investments in medical schools, hospitals, clinics, labs, libraries, and research institutes are among them. Enhancing collaboration and coordination among medical education institutions, health authorities, professional groups, and international organizations to exchange best practices, standards, curriculum, professors, and students is another approach [9,10]. A third approach is to encourage innovation and entrepreneurship in medical education by establishing centers, programs, and platforms that promote innovative teaching and learning methodologies, digital health technology, and social impact projects [7,10]. These solutions attempt to enhance the quality and results of medical education in the Arab world, as well as to address the population's present and future health demands.

Conclusion

Medical education in the Arab world has several problems, particularly in light of social, political, and economic developments. The coronavirus disease 2019 (COVID-19) epidemic has highlighted both the region's strengths and inadequacies in medical education. As a medical student in the Arab world, I wanted to express some of my thoughts on the present situation and potential solutions.

Despite these challenges, there are several possibilities and strengths that may be used to improve medical education in the Arab world. One of them is the availability of technology and digital platforms that may help with information access, communication, cooperation, and creativity. The COVID-19 pandemic has hastened the adoption of online learning and telemedicine, which have the potential to improve the quality and efficiency of medical education and practice. However, it is critical to guarantee that these technologies are used correctly, ethically, and successfully.

Medical education in the Arab world is facing a critical era of development that will need collaborative efforts from every group involved to solve problems and capitalize on possibilities. As a regional medical student, I hope that this article has thrown some light on some of the challenges that impact our education and future professions. I also hope that it has sparked some conversation and action among educators, politicians, practitioners, and students in our region to enhance medical education.
